# Inferring explicit weighted consensus networks to represent alternative evolutionary histories

**DOI:** 10.1186/1471-2148-13-274

**Published:** 2013-12-23

**Authors:** Mehdi Layeghifard, Pedro R Peres-Neto, Vladimir Makarenkov

**Affiliations:** 1 Département des Sciences biologiques, Université du Québec à Montréal (UQÀM), CP 8888, Succ. Centre Ville, Montréal, QC H3C 3P8, Canada; 2 Département d’Informatique, Université du Québec à Montréal (UQÀM), CP 8888, Succ. Centre Ville, Montréal, QC H3C 3P8, Canada

**Keywords:** Consensus network, Consensus tree, Phylogenetic network, Phylogenetic tree, Reticulate evolution

## Abstract

**Background:**

The advent of molecular biology techniques and constant increase in availability of genetic material have triggered the development of many phylogenetic tree inference methods. However, several reticulate evolution processes, such as horizontal gene transfer and hybridization, have been shown to blur the species evolutionary history by causing discordance among phylogenies inferred from different genes.

**Methods:**

To tackle this problem, we hereby describe a new method for inferring and representing alternative (reticulate) evolutionary histories of species as an explicit weighted consensus network which can be constructed from a collection of gene trees with or without prior knowledge of the species phylogeny.

**Results:**

We provide a way of building a weighted phylogenetic network for each of the following reticulation mechanisms: diploid hybridization, intragenic recombination and complete or partial horizontal gene transfer. We successfully tested our method on some synthetic and real datasets to infer the above-mentioned evolutionary events which may have influenced the evolution of many species.

**Conclusions:**

Our weighted consensus network inference method allows one to infer, visualize and validate statistically major conflicting signals induced by the mechanisms of reticulate evolution. The results provided by the new method can be used to represent the inferred conflicting signals by means of explicit and easy-to-interpret phylogenetic networks.

## Background

Molecular data have played an instrumental, and usually indispensable, role in many phylogenetic and evolutionary studies in the recent decades. Their increasing availability is due to outstanding advances in the development of fast, efficient and affordable sequencing technologies [1]. Although this growth has triggered the advancements of theoretical informatics aspects of phylogenetics and evolutionary biology via the development of new algorithms, statistical models and software, fast and effective analytical methods have yet to be designed to take advantage of this huge surplus of data. For instance, the field of phylogenetics still faces some key analytical challenges stemming from reticulate evolution. They include: 1) horizontal gene transfer (e.g., in bacterial or viral evolution); 2) hybridization among species (e.g., allopolyploidy in plants); 3) genetic differentiation of allopatric populations and gene exchange through migration; 4) homoplasy (i.e., parallel evolution and reversals); 5) incomplete lineage sorting; and 6) recombination between genes [2-5]. All these processes may lead to the incongruity among gene trees [6-10] inferred from the data affected by reticulate evolutionary mechanisms. Implicit or explicit phylogenetic networks should be used to represent these complex phenomena when the gene tree incongruity is observed [5,11]. Implicit networks are better suited for a general representation of conflicting evolutionary signals present in the data, whereas explicit networks are used for depicting the precise reticulation events, including their directionality and the species involved. The inference and validation of explicit phylogenetic networks is the main goal of the current study.

Another key factor that contributes to the incompatibility among gene trees is stochastic errors resulting from analytical features such as choice of optimality criterion, taxon sampling and sequence evolution model [12-14]. These complications not only makes it difficult for researchers to find reliable estimates of the true species phylogenies, but also obstruct such fields as comparative biology and community phylogenetics which rely on phylogenetic trees in their analyses [15-17].

Evidence from many studies conducted on different groups of species, from fruit flies to hominids [10,18-25], have shown that gene tree discordance is a widespread phenomenon. These studies mostly concluded that rarely a predominate or consistent single-gene-based phylogeny could be perceived or reconstructed for a moderate to large set of species, regardless of the type of phylogenetic data at hand. Among the traditional tree-like techniques developed to solve the gene tree incongruence problem there are two widely used approaches of gene concatenation and consensus tree reconstruction, both of which result in the inference of a single phylogenetic tree as the most probable representation of the evolutionary history of species.

Although there have been successful cases of using the *concatenation approach* to elucidate the ancestral relationships among certain groups of species [26-29], multi-gene datasets very rarely converge to the same phylogeny, more often providing results which are contradictory or inconsistent with well-known and highly reliable species trees [7,30-32]. These statistical inconsistencies in estimating phylogenetic trees using concatenated datasets have been confirmed by simulation studies [33,34].

The main idea behind traditional *consensus tree* reconstruction methods is that each of the phylogenetic trees from a given collection of trees should contribute to a consensus tree according to the presence of its clusters. Among the most known and widely used consensus tree reconstruction methods are the majority rule consensus [35] and Nelson (often called Nelson-Page) consensus approaches [36,37]. The traditional strict majority rule consensus tree includes all the clusters that occur in more than 50 % of the considered trees. The major pitfall of this method is that for a set of trees with a poor overall bootstrap support, the 50 % cluster occurrence constraint leads to a very weakly resolved phylogeny. On the other hand, in the extended majority rule consensus tree approach, a strict consensus tree is first constructed and then the remaining compatible clusters are added to it following their overall frequency in the considered tree collection. For the collections of trees with a poor overall bootstrap support, the constraint of 50 % used when inferring the majority rule and extended majority rule consensus trees can be often inconvenient. Many existing software allow for clusters that are present in less than 50% of the trees. They work downwards in the frequency of the cluster occurrences as long as the new clusters aid to resolve the consensus tree. The extended majority rule consensus method often provides solutions similar to those of the Nelson consensus method, although not necessarily identical to them [36,37]. The Nelson consensus method, first described in [36] and then generalized in [37], relies on the graph theory techniques to find maximum cliques of mutually compatible clusters. Its major drawback is that these cliques do not always contain enough compatible clusters to constitute a fully resolved phylogenetic tree [38]. Moreover, the problem of finding a maximum clique of compatible clusters has been shown to be NP-hard [39].

Phylogenetic networks should be used instead of consensus species trees or species trees inferred from concatenated sequences whenever reticulate evolutionary processes are studied [3,5,11]. Here, we recall some of the existing phylogenetic network building methods and software based on the cluster support. In an early attempt to build *consensus phylogenetic networks*, Holland et al. [40] developed an implicit consensus network model based on the median network method [41] to visualize incompatibilities encompassed in the given collection of trees. This method proceeds first by ranking all the splits according to their frequency and then builds a system of compatible splits by adding those splits to the network, one at a time, following their frequency ranking. Holland and colleagues [42] further optimized their original greedy consensus network method to incorporate weights from individual trees into the network inference process. Having the length of each split (i.e., branch length of the split branches) in different trees as well as the weights associated with those trees, this method computes an average length for each split and finally selects compatible splits based on their weights to build a consensus network.

In another attempt, Huson [43] and then Huson and Bryant [4] have developed a computer program called *SplitsTree* which reconstructs an unrooted splits graph from a collection of phylogenetic trees through selecting all the splits that are present in more than a fixed percentage of all the trees [40]. However this program provides as result only implicit network structures, the inferred extra links do not usually directly correspond to the tree lineages and the number of nodes and edges of the resulting network can grow exponentially with the number of splits. To address these disadvantages, Huson and Rupp [44] proposed the *cluster network* approach to build a phylogenetic network from a collection of gene trees using a modified *tree popping algorithm* which they called *network popping algorithm*. To estimate the support of any reticulation edge, the average support of that edge (computed over all trees) is divided by the average support of the alternative reticulation edges located at the same position and weighted by the average support of all other tree edges [5,44]. The latter authors stated however that no association between clusters and reticulation edges is provided by this method. For instance, the obtained cluster support was not shown in their network representations [44]. On the other hand, Abby et al. [45] proposed a horizontal gene transfer inference method called *Prunier*. Prunier needs a species tree and a gene tree as a reference and does not treat multiple gene trees. Prunier relies on a ranking of branches that are common to the species and gene trees based on the amount of conflicts that is reduced when the branch is removed. This amount of conflicts is a function that depends on the statistical support of the internal branches of the gene tree. For a detailed review of the existing phylogenetic network reconstruction methods the reader is referred to [5]. Mention that the results yielded by most of the existing consensus network building methods are implicit and generally not easy to interpret.

In this study, we present a new algorithm for the inference of *explicit weighted consensus networks* from a collection of trees (e.g., multiple single-gene phylogenies), with or without prior knowledge of the species phylogeny. Such networks are capable of representing the main historical pattern of the species evolution (i.e., associated with the clusters present in the species tree) as well as the alternative evolutionary routes characterizing the species and genes under consideration. The main advantage of our method is that it allows for visualizing the species evolutionary relationships in a very clear and easy-to-interpret manner. Our algorithm takes advantage of the weights (e.g., least-square scores, posterior probabilities, maximum likelihood scores or *p*-values) assigned to the gene trees as well as the weights associated with the tree clusters (e.g., cluster's bootstrap score or posterior probability) to infer the species dominant and alternative evolutionary histories. If a species tree is provided in addition to the collection of gene trees, our algorithm considers it as the dominant evolutionary history (i.e., backbone structure) and uses the collection of gene trees to infer the most significant reticulation events. If only a collection of gene trees is given, the new algorithm first builds a weighted consensus tree as the main evolutionary pattern and then infers the most significant alternative events.

The rest of the article is organized as follows. In the Methods section, a description of the basic concepts of phylogenetic networks and a detailed presentation of our new algorithm are given, followed by the description of the simulation protocol and the three considered real datasets. In the Results section, the results and performances of the new algorithm obtained for both simulated and real data are reported. They are then discussed in detail in the final section of the article.

## Methods

### Basic concepts

#### Graph

A graph *G* (*V*, *E*) consists of a collection of vertices (*V*) which are connected by a collection of edges (*E*) in a pairwise manner. A path in a graph is a sequence of at least two vertices (*v*_1_, *v*_2_, …, *v*_*k*_) such that, for all *i* ∈ {1, 2, …, *k*-1}, there exists an edge {*v*_*i*_, *v*_*i*+1_} in *E*. A cycle in a graph is a path whose first and last vertices are the same, while all other edges and vertices are pairwise distinct.

### Phylogenetic tree

A *phylogenetic tree* (*T*) is an acyclic connected graph whose leaves (i.e., vertices of degree one) are labelled according to the given set of taxa (i.e., species). Phylogenetic trees can be either bifurcating (i.e., all the internal nodes have an indegree of one and an outdegree of two) or multifurcating (i.e., internal nodes can have an outdegree of three and more). Phylogenetic trees can be rooted or unrooted, where the root is a node representing a common ancestor of all the species involved in the analysis.

### Phylogenetic network

A *phylogenetic network* is a connected graph used either to visualize evolutionary relationships between species or to display conflicting evolutionary signals without such limitations as being acyclic or having a fixed indegree or outdegree of its nodes. Phylogenetic networks can be implicit or explicit: implicit networks such as split graphs are used to represent conflicting and ambiguous signals in a dataset using parallel sets of edges, rather than single branches. These networks often contain nodes that are not representing any ancestral species, hence providing only an *implicit* representation of evolutionary histories [4]. In explicit networks, in contrast, the internal nodes represent ancestral species and nodes with more than two parents correspond to reticulation events such as hybridization, recombination or horizontal gene transfer. Such networks provide an *explicit* representation of evolutionary history of species (see [5] for more details).

Here, we will first define some basic principles of the weighted consensus tree reconstruction prior to expanding them to phylogenetic networks inferring.

### Bootstrap-based majority rule consensus tree

**Figure 1:**
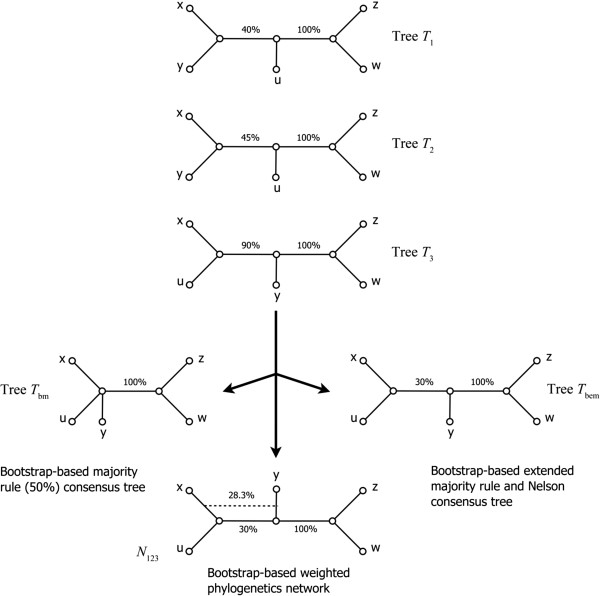
**Bootstrap**-**based consensus trees and networks.** Bootstrap-based majority rule consensus tree *T*_*bm*_, bootstrap-based extended majority rule consensus tree *T*_*bem*_ and weighted implicit phylogenetic network *N*_*123*_ for a collection of three binary phylogenetic trees *T*_1_, *T*_2_ and *T*_3_ whose leaves are labelled by the set of 5 taxa (*x*, *y*, *z*, *w* and *u*). The bootstrap scores of the internal branches of trees *T*_1_, *T*_2_ and *T*_3_ are indicated. All the trees have the same weight.

The main idea of our approach is that each phylogenetic tree from a given collection of trees should contribute to a consensus tree not simply by the presence, but also by the quality of its clusters (i.e., bipartitions or splits corresponding to the internal tree branches). The quality of a cluster within a given collection of trees can be defined as the sum of bootstrap scores, taken over all the trees in this collection, of the internal branches associated with this cluster. The traditional majority rule consensus tree includes only the clusters that exist in more than 50% of the considered trees [35]. Note that any other percentage between 50% and 100% can also be specified in most of the existing phylogenetic packages (e.g., in PHYLIP [46]). The *bootstrap*-*based majority rule consensus tree* will include any cluster whose average bootstrap support, i.e., total sum of bootstrap scores, computed over all the trees in the collection, divided by the number of trees in this collection, is greater than 50% (e.g., tree *T*_*bm*_ in Figure 1 is the bootstrap-based majority rule consensus tree, as well as the strict majority rule consensus tree, of trees *T*_1_, *T*_2_ and *T*_3_). It is easy to prove that all the clusters satisfying such a rule will be pairwise compatible. For this, it will be sufficient to substitute each tree of the original tree collection by the set of its bootstrap replicates (i.e., replicated trees built when carrying out the bootstrap procedure) and then apply the traditional strict majority rule method on this extended set of replicated trees. All the clusters appearing in more than 50% of the replicated trees will be mutually compatible.

### Bootstrap-based extended majority rule consensus tree

Similar to the traditional extended majority rule method, as implemented in the CONSENS program of the PHYLIP package [46], the *bootstrap*-*based extended majority rule* method is a two-stage procedure. First, any cluster whose average bootstrap score is greater than 50% will be included in the consensus tree. Then, the method will consider the remaining clusters following the order of their total sums of bootstrap scores, computed over all the trees in the collection, and gradually add to the consensus tree those that are compatible with the current consensus tree until the tree is fully resolved or no more compatible clusters remains. For instance, tree *T*_*bem*_ in Figure 1 is the extended bootstrap-based majority rule consensus tree of trees *T*_1_, *T*_2_ and *T*_3_.

### Bootstrap-based Nelson consensus tree

We also consider the following extension of the traditional Nelson method. To build the *bootstrap*-*based Nelson consensus tree* each clique will be assigned a score equal to the sum of scores of clusters included in it. The score of each cluster is defined as a sum of bootstrap scores associated with this cluster, computed over the given collection of trees. Unlike the method described by Page [37], where only the replicated clusters can contribute to the clique scores, our procedure also takes into account the scores of all unreplicated clusters. If a single clique with the highest total bootstrap score is found, the group of compatible clusters included in this clique will define the bootstrap-based Nelson consensus tree. If there exist more than one such clique, then the bootstrap-based Nelson consensus tree will contain only the clusters found in all of the maximal replication cliques. In this case, clusters found in some, but not all, of the maximal-replication cliques can be classified as “ambiguous” (for more details see [37,46,47]). In some cases, the bootstrap-based extended majority tree and Nelson consensus tree will be identical (e.g., tree *T*_*bem*_ in Figure 1 is also the Nelson consensus tree of trees *T*_1_, *T*_2_ and *T*_3_), but this equivalence does not hold in general.

In Figure 1, a set of three trees is presented (*T*_1_, *T*_2_ and *T*_3_), each of them containing two internal branches with the associated bootstrap scores. The right-hand internal branch (connecting leaves “*z*” and “*w*” to the rest of the tree) has bootstrap support of 100% in all three trees. Therefore, it should be included in all consensus trees, or networks, regardless of the reconstruction method used. On the other hand, the left-hand internal branch connecting leaves “*x*” and “*y*” to the rest of the tree in *T*_1_ and *T*_2_ has different bootstrap scores in these trees (40 and 45% respectively). In tree *T*_3_, the left-hand internal branch connects leaves “*x*” and “*u*” to the rest of the tree. Its bootstrap score, 90%, is higher than the sum of bootstrap scores of the corresponding branch in *T*_1_ and *T*_2_. When using the bootstrap-based majority rule defined above, we obtain a consensus tree (*T*_*bm*_ in Figure 1) that does not include the left-hand internal branch because neither the sum of scores of *T*_1_ and *T*_2_ nor the bootstrap score of *T*_3_ divided by the number of trees is greater than 50%. The application of the bootstrap-based extended majority rule adds to the consensus tree (tree *T*_*bem*_ in Figure 1) the left-handed branch of tree *T*_3_, since 90% / 3 = 30% > (40% + 45%) / 3 = 28.3%. Tree *T*_*bem*_ is also the bootstrap-based Nelson consensus tree of *T*_1_, *T*_2_ and *T*_3_. Finally, the construction of the bootstrap-based consensus network (*N*_123_ in Figure 1) relies on the same principle as the bootstrap-based extended majority rule, except that it encompasses both left-hand internal branches (that from *T*_1_ and *T*_2_ and that from *T*_3_) characterized by their bootstrap support. Network *N*_123_ is an implicit consensus network. In this article we will show how such an implicit network can be transformed into explicit one depending on the evolutionary mechanism being studied.

### Method description: consensus tree

The method we present and apply here also takes into consideration the weights associated with the given phylogenetic trees in addition to bootstrap scores of the tree clusters (i.e., internal branches). Using one of the three equations presented in the section “Inferring weights”, the method defines a weight of each cluster based on the weights of the trees containing this cluster and on the cluster’s bootstrap scores in these trees. Then, after ranking all the clusters based on their weights, it regroups the compatible clusters starting from the top of the list, until a fully resolved consensus tree is built. This method is called here *weight*-*based extended majority rule consensus tree inference*.

### Method description: consensus network

Our *consensus network inference method* accepts two types of input: 1) a species phylogenetic tree and a set of gene phylogenetic trees defined on the same set of species, or 2) only a set of gene trees defined on the same set of species. In phylogenetic studies, gene trees are usually characterized by their weights that reflect the quality of the reconstruction process. Such weights could be an average of bootstrap scores of the tree’s internal branches, a maximum parsimony or maximum likelihood score or a Bayesian posterior probability estimate. Thus, we assume that all the phylogenies have bootstrap scores or posterior probabilities (or any other measure of support) for their internal branches. Our algorithm first breaks down all the gene phylogenies into their relevant clusters and calculates a weight for each cluster based on Equations 1, 2 or 3 presented in the following section. Next, the algorithm ranks all the clusters based on their weights. For this type of input, our algorithm uses the species tree as the backbone of the network and gradually adds to it the highly ranked clusters (i.e., represented by reticulation branches) of the gene phylogenies. For the first type of input, the species tree is accepted as the dominant evolutionary history and the clusters of the gene trees are used to infer the reticulate (alternative) evolutionary events. For the second type of input, our algorithm reconstructs a consensus phylogenetic tree using the weight-based extended majority rule consensus tree method described above and then adds to it the remaining highly ranked incompatible clusters which are presented as reticulation branches. In the obtained consensus network, the weight-based consensus tree and the reticulation branches can be regarded as the main and alternative evolutionary scenarios, respectively.

**Figure 2:**
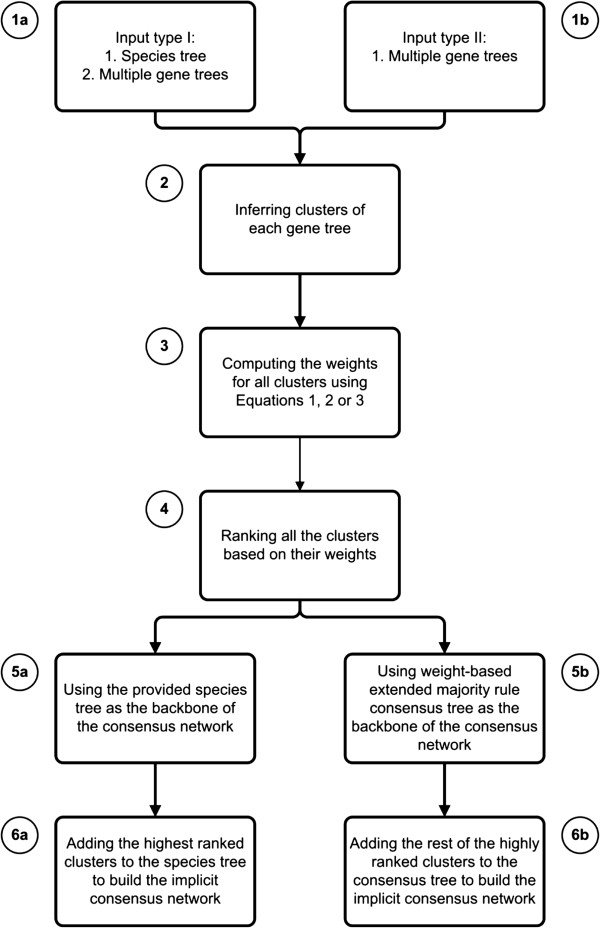
**Flowchart of the new method for building weighted consensus networks.** Facet *a* of the method (indicated by lowercase *a* next to step numbers) uses a species tree as well as a set of gene trees to infer the consensus network. Facet *b* of the method (indicated by lowercase *b* next to step numbers) uses only a set of gene trees to build the consensus network. Step numbers that do not contain any letter are common steps for the two facets.

Regardless of the input type, the resulting representation is a weighted consensus phylogenetic network with a backbone tree structure and reticulation branches being chosen based on their weights which reflect their contribution to the clustering process. These two algorithmic facets are schematically presented in Figure 2, in which the steps depicted by letter *a* correspond to processing the first type of input and those depicted by letter *b* are related to the second type of input. Steps 2 to 4 are common for both types of input.

We present here three network building algorithms (Algorithms 1, 2 and 3), each of them being optimized for detecting and representing a specific evolutionary phenomenon. The first algorithm (Algorithm 1), which accepts the input of type 2 (a collection of gene trees inferred for various genes), is suitable for inferring either diploid or polyploidy hybridization events occurred among the observed species, or for finding recombination events occurred at the chromosome level. Algorithm 1 first proceeds by building the weight-based extended majority rule consensus tree followed by finding reticulation events and adding them to the consensus tree with proper direction in order to build the explicit weighted consensus network. The time complexity of Algorithm 1 is *O*(*n* × *m*^2^ × (*n* + *r*)), where *n* is the number of gene trees in the considered gene tree collection τ, *m* is the number of leaves in each of these trees and *r* is the number of reticulation branches (i.e., reticulation events) added to the consensus tree. Note that the cluster inference procedure in Algorithm 1 (i.e., the first loop *for* in this algorithm) has the time complexity of *O*(*n* × *m*^2^) as we use an optimal algorithm for the tree cluster inference, originally described by Makarenkov and Leclerc [48], in which each tree cluster is presented as a binary bipartition vector. The weight computation procedure for the clusters from the gene tree collection τ (i.e., the second loop *for* in Algorithm 1) has the time complexity of *O*(*n*^2^ × *m*^2^). The time complexity of the second loop *while* in this algorithm, where the reticulation branches are added to the consensus tree, is *O*(*r* × *n* × *m*^2^). The function *find*_*direction* in the same algorithm has the time complexity of *O*(*n* × *m*^2^). A group of clusters (i.e., bipartition vectors) is called *compatible* if altogether these clusters induce a unique phylogenetic tree. A cluster *c* has the *first degree of incompatibility* with a phylogenetic tree *T* if there exists an SPR (Subtree Prune and Regraft) move of the branches of *T* induced by the cluster *c* that transforms *T* into another phylogenetic tree. For instance in Figure 1, cluster (*xy*) has the first degree of incompatibility with tree *T*_3_. In the same way, cluster (*xyw*) has the second degree of incompatibility with tree *T*_3_, as it requires two SPR moves (i.e., two reticulation branches) to transform *T*_3_ into a tree where cluster (*xyw*) is present. In the case of a directed phylogenetic network *N*_*h*_ inferred in Algorithm 1, cluster *c* will have the first degree of incompatibility with *N*_*h*_ if it has the first degree of incompatibility with the tree *T* obtained from *N*_*h*_ after carrying out all SPR moves corresponding to the reticulation branches included in *N*_*h*_. Mention that in all the three presented algorithms we only need to know whether a given cluster *c* has the *first degree of incompatibility* with a given network *N*_*h*_ or not.

Algorithm 2, on the other hand, is designed to infer intragenic recombination events or partial horizontal gene transfers which lead to the creation of mosaic genes. This algorithm accepts two types of input (a species tree and a multiple sequence alignment, or only a multiple sequence alignment). In cases where a species tree is provided, Algorithm 2 uses it as a backbone of the network. A sliding window procedure is then carried out for finding the aforementioned reticulation events and adding them to the backbone in order to build an explicit weighted consensus network. Otherwise, if only a multiple sequence alignment is given, a weight-based extended majority rule consensus tree will be built from it and used as the backbone of the network. The time complexity of Algorithm 2 is *O*(|*SW*| × (*O*(*PhylInfMeth*) + *n* × *m*^2^ × (*n* + *r*))), where |*SW*| is the cardinality of the set of *MSA* (multiple sequence alignment) fragments examined by the sliding window procedure and *O*(*PhylInfMeth*) is the running time of the phylogeny inference method used to infer the tree *T* from the *MSA* fragment *MSA*_*f*_.

Our third algorithm (Algorithm 3) is intended for finding complete horizontal gene transfer events. It accepts as input a species tree in addition to one or more gene trees (or multiple sequence alignments). Algorithm 3 uses the species tree as the backbone for the network and adds to it the most significant clusters (i.e., horizontal gene transfer events) obtained after computing the weights of the gene tree clusters in order to build the weighted consensus horizontal gene transfer network. The time complexity of Algorithm 3 is *O*(*PhylInfMeth*) + *O*(*n* × *m*^2^ × (*n* + *r*)).

**Figure 3:**
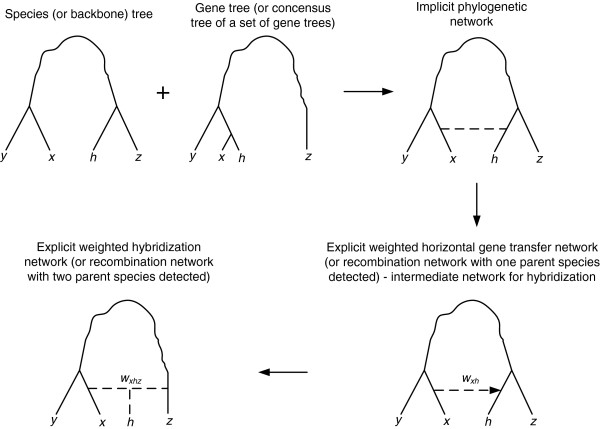
**Building explicit weighted consensus phylogenetic networks.** The explicit network is built from sets of clusters defined by a species (i.e. backbone) phylogenetic tree and a gene tree (or a set of gene trees): An implicit weighted phylogenetic network is first constructed; then, it is transformed into an explicit weighted horizontal gene transfer network, which can be transformed into an explicit hybridization network. Traditional (i.e. complete) horizontal gene transfer, partial horizontal gene transfer and recombination events for which the recombinant organism and only one of its parents can be identified give rise to a horizontal gene transfer network. Diploid and polyploid hybridization along with recombination events for which the recombinant organism and both of its parents can be identified give rise to a hybridization network. Straight lines indicate single tree or network branches, dashed lines - reticulation branches and wavy lines - paths including multiple branches.

The resulting phylogenetic network, regardless of the algorithm used, will be an explicit (in the sense that it represents exactly the assumed evolutionary mechanism) weighted and directed consensus network as shown in detail in Figure 3. The weight estimates of the obtained backbone and reticulation branches provide statistical support of the inferred speciation and reticulation events.

### Inferring cluster weights

For each cluster from the set of the given gene trees, we have first to compute its overall weight. Every tree cluster can be associated with two types of initial weights, one being its proper bootstrap score or posterior probability in its tree of origin and another characterizing its entire tree of origin. In the case when the input contains only the weights associated with internal tree branches and lacks any measure of support for entire trees, we use the following equation to calculate the overall cluster weights:

(1) $$W_{i}\left(C\right)=\left(\sum_{j=1}^{n}\sigma_{\mathrm{ij}}\times W\left(C_{\mathrm{ij}}\right)\right)/n,$$

where *W*_*i*_(*C*) is the overall weight of cluster *i*, *W*(*C*_*ij*_) is the weight of cluster *i* in tree *j* and *n* is the total number of trees. If cluster *i* is absent in tree *j*, then *σ*_*i*_ equals 0, otherwise it equals 1.

Conversely, when the entire tree support is provided for each tree from the given set of trees but the input lacks individual supports for internal branches, we use the following equation to calculate the overall cluster weights:

(2) $$W_{i}\left(T\right)=\left(\sum_{j=1}^{n}\sigma_{\mathrm{ij}}\times W\left(T_{j}\right)\right)/n,$$

where *W*_*i*_(*T*) is the overall weight of cluster *i* calculated from the tree supports only, *W*(*T*_*j*_) is the support of tree *j* and *n* is the total number of trees.

Finally, when both cluster and tree initial supports are provided in the input, we use the following equation to infer the overall cluster weight, *W*_*i*_(*C*,*T*), for each cluster *i*:

(3) $$W_{i}\left(C,T\right)=\left(\sum_{j=1}^{n}\sigma_{\mathrm{ij}}\times W\left(C_{\mathrm{ij}}\right)\times W\left(T_{j}\right)\right)/n,$$

where *W*(*C*_*ij*_) is the weight of cluster *i* in tree *j*, *W*(*T*_*j*_) is the support for tree *j* and *n* is the total number of trees. These overall cluster weights will be used to build the consensus tree or network as described above.

### Assessing the efficiency of the new method

#### Real data

We examined three evolutionary datasets to test the efficiency of our weighted consensus network inference method. The first dataset consisted of 677 bp nucleotide sequences of mitochondrial *cytochrome c oxidase subunit II* of six species of honeybees (subfamily Apinae). The second one comprised eight chloroplast 16S rRNAs (920 nucleotides) from plants, algae and cyanobacterium. These two datasets are well-known and distributed with the SplitsTree program [43] among the data encompassing the events of reticulate evolution. The third considered dataset consisted of amino acid sequences of ribosomal protein *rpL12e* for 14 Archaeal species [49].

We applied four different tree inference methods on both real and simulated (described in the next section) data to produce collections of gene trees. One representative from each of the four main tree reconstruction approaches (i.e., distance-based [50], maximum parsimony [51], maximum likelihood [52] and Bayesian [53] approaches) was considered. The exact methods we used were the following: BIONJ [54], DNAPARS from the PHYLIP package [55], PhyML [56] and MrBayes [57].

We applied these tree inference methods on both whole sequences and fragments of sequences (using a sliding window procedure) in order to search for alternative evolutionary events which might have affected either entire gene sequences (e.g., hybridization events) or only small sequence fragments (e.g., partial horizontal gene transfer events). The latter events are usually ignored when analyzing entire genetic sequences during tree or network reconstruction. In the case of horizontal gene transfer events in Archaebacteria, we also computed the directions of complete and partial horizontal gene transfers using a dedicated function based on the Robinson and Foulds topological distance [58]; see the function *find*_*direction* in the end of Algorithm 1. Assume that *T* is the backbone phylogenetic tree and *r* is the newly found horizontal gene transfer event between clusters *C*_1_ and *C*_2_ (i.e., groups of species related by *r*). Let *T*_1_ be the tree obtained by an SPR move induced by reticulation branch *r* with direction *d*_1_ (corresponding to the horizontal gene transfer from cluster *C*_1_ to cluster *C*_2_) and *T*_2_ be the tree with *r* added to represent the gene transfer in the opposite direction (i.e., from *C*_2_ to *C*_1_). Then, the cumulative Robinson and Foulds distance is calculated between *T*_1_ and all the original gene trees containing cluster *C* = *C*_1_ ∪ *C*_2_, on one hand, and *T*_2_ and all the original gene trees containing *C*, on the other hand. Finally, the obtained cumulative Robinson and Foulds distances are weighted by the support of the original gene trees containing *C* as it is shown in Algorithm 1 (see the exact formula is in the function *find*_*direction*) and the resulting inequality indicates the direction of the horizontal gene transfer *r*.

#### Simulated data

We generated sets of trees encompassing multiple reticulation features to test the efficiency of the proposed consensus network inference method in the context of recombination. First, random binary phylogenetic trees were generated using the procedure originally described by Kuhner and Felsenstein [59]. The branch lengths of these phylogenies were computed using an exponential distribution. Following the approach of Guindon and Gascuel [60], we added some noise to the tree branches to create a deviation from the molecular clock hypothesis. All branch lengths were multiplied by 1 + *ax*, where the variable *x* was obtained from an exponential distribution (*P*(*x* > *k*) = exp (−*k*)), and the constant *a* was a tuning factor accounting for the deviation intensity. The value of *a* was fixed to 0.8. The random trees generated by this procedure had depth of *O*(log (*n*)), where *n* was the number of species (i.e., number of leaves in a binary phylogenetic tree).

Second, we ran the SeqGen program [61] to generate DNA sequences along the branches of the phylogenies constructed at the first step. SeqGen was used with the HKY model of nucleotide substitution, model of rate heterogeneity assigning different rates to different sites according to a gamma distribution (with the shape parameter equal to 1.0) and (TS/TV) ratio equal to 2.0. These settings were selected in order to render the simulation parameters similar to those used when processing the real datasets. The DNA sequences with 400 nucleotides were generated. Third, using the reticulation events generation procedure described in [62], we incorporated the blocks of fragments induced by recombination into the generated multiple sequence alignments (MSAs). The sliding window procedure was then employed to recover these recombined blocks of sequences. Forth, for each generated MSA, the BIONJ, DNAPARS, PhyML and MrBayes methods were carried separately to infer phylogenetic trees for the whole MSA and for each MSA fragment corresponding to the fixed position of the sliding window. Finally, we carried the proposed weighted consensus network building method to infer the consensus tree topology (i.e., backbone evolutionary structure representing the most significant speciation events) as well as to recover the most significant (those with the highest weights) recombination events. We repeated this procedure 100 times for each original tree, i.e., 100 different MSAs were generated for the same original tree. The sliding window sizes considered in our simulations were 10, 20, 30, 40 and 50% of the total length of the generated MSAs. The sliding window progress step of 5 nucleotides was adopted. Simulations were carried out with the phylogenies having 16, 24, 32, 48 and 64 leaves and encompassing 1 to 8 recombination events.

## Results

### First example: honeybee data

**Figure 4:**
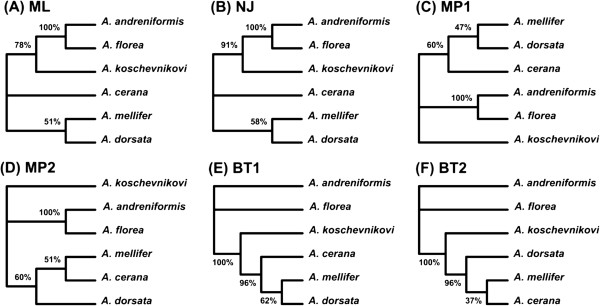
**The set of six gene trees (A-F) obtained using different tree reconstruction methods for honeybee dataset.** ML, NJ, MP and BT abbreviations stand for trees obtained by maximum likelihood, neighbour-joining (here a distance-based approach implemented in BIOINJ), maximum parsimony and Bayesian approaches, respectively.

We applied the BIONJ, DNAPARS, PhyML and MrBayes methods to infer the evolutionary history of the six honeybee species. The inferred trees are shown in Figure 4. The BIONJ and PhyML methods provided a single phylogeny (Figures 4A and 4B, respectively). In contrast, two optimal phylogenies were obtained by each of the DNAPARS and MrBayes methods (Figures 4C and 4D represent maximum parsimony trees and Figures 4E and 4 F represent Bayesian trees). For the sake of simplicity, we assigned a total weight of 1 to each of the considered methods. Therefore, the BIONJ and PhyML phylogenies received a weight of 1, whereas each of the DNAPARS phylogenies received a weight of 0.5. For the case of Bayesian phylogenies, we also used their specific posterior probabilities whose sum was scaled to 1.

**Figure 5:**
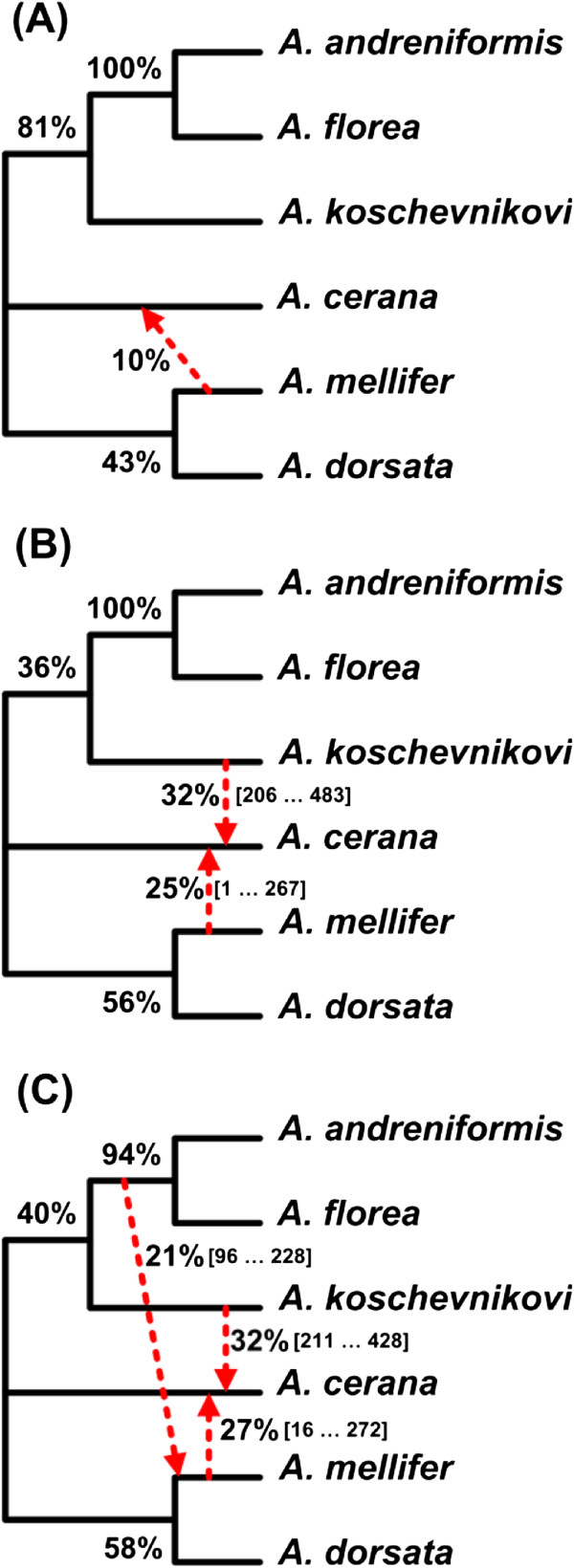
**Explicit weighted consensus networks inferred for the honeybee dataset. A)** network obtained from full-length sequences using all the six trees from Figure 4 (which were inferred using the ML, NJ, MP and BT approaches); **B)** network obtained by the sliding window procedure with a ML method used for tree inference; **C)** network obtained by the sliding window procedure with a Bayesian method used for tree inference. The bootstrap scores of internal branches of the backbone tree and the weights of reticulation branches are indicated. The sliding window procedure was used to detect smaller-scale reticulation events which are represented by dashed lines in parts **B** and **C** of the figure. For each small-scale event, the sequence interval corresponding to this event is given between brackets.

After breaking down the phylogenies into their clusters and calculating the cluster weights using Equation 3, we ranked all the clusters according to their weights and put together the compatible clusters to build the backbone of the consensus network based on the clusters ranks. Finally, we added the rest of the highly ranked clusters to the backbone tree to construct a weighted consensus network of the six honeybee species. In this analysis, we found one reticulation branch (alternative event) in addition to the backbone (consensus tree). The explicit weighted consensus network built using Algorithm 1 is shown in Figure 5A. It depicts one recombination event which might have influenced the evolution of the considered honeybee species.

### Second example: chloroplast data

**Figure 6:**
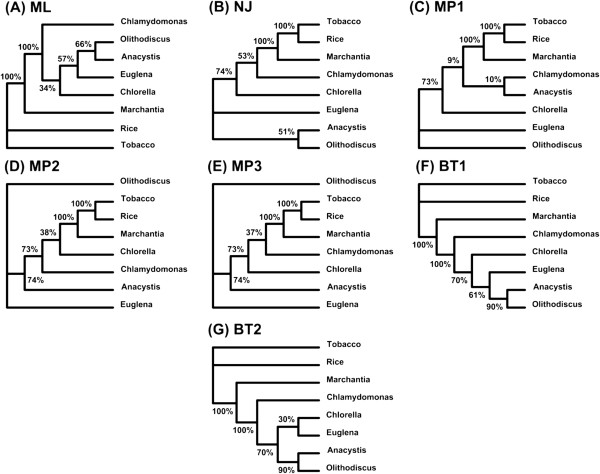
**The set of seven gene trees (A-G) inferred for the chloroplast dataset.** The abbreviations used in Figure 4 also apply here.

In this example, we used the same four tree inference methods as in the previous section to model evolutionary relationships among the eight plants from the chloroplast dataset. The application of these methods resulted in one maximum likelihood (Figure 6A), one distance-based (Figure 6B), three maximum parsimony (Figures 6C to 6E) and two Bayesian phylogenies (Figures 6F and 6G). Similar to the previous example, we assigned a total weight of 1 to each method. Therefore, the BIONJ and PhyML phylogenies received the weight of 1 while each of the DNAPARS trees received the weight of 0.33. In the case of the MrBayes phylogenies, we also used their corresponding posterior probabilities scaling their sum to 1.

We, then, computed the weights of all the clusters presented in at least one of the seven phylogenetic trees using Equation 3. Finally, we built the backbone of the consensus network and added to it the reticulation branches after ranking the clusters as described in Algorithm 1.

**Figure 7:**
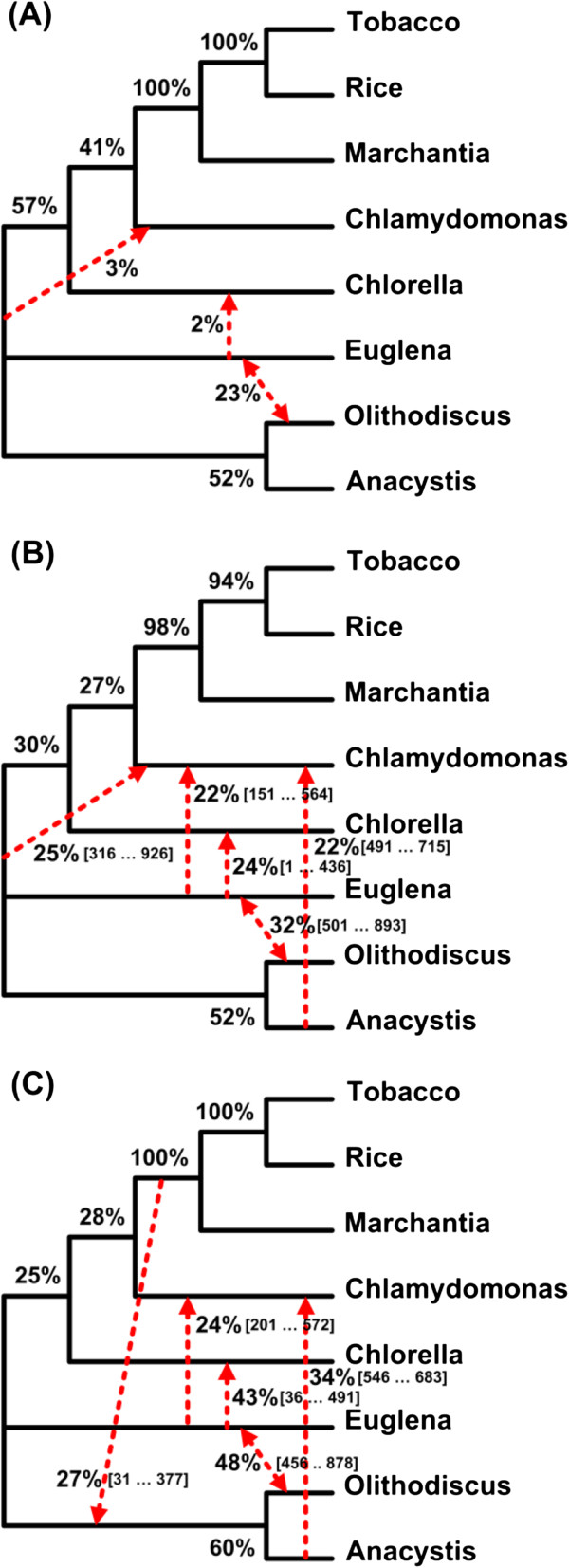
**Explicit weighted consensus networks obtained for the chloroplast dataset. A)** network obtained from full-length sequences using all the seven trees from Figure 6 (which were inferred using the ML, NJ, MP and BT approaches); **B)** network obtained by the sliding window procedure with a ML method used for tree inference; **C)** network obtained by the sliding window procedure with a Bayesian method used for tree inference. The notations of Figure 5 also apply here.

In this analysis, we found three reticulation branches which represent possible recombination events. The reconstructed weighted consensus network of the plastid 16 s rRNAs is shown in Figure 7A. Using the cut-off level of 10% and eliminating the two poorly supported reticulation branches (those with the weights of 2% and 3%) would provide us with the weighted consensus network encompassing one probable reticulation event only (that with the weight of 23%).

### Third example: archaebacteria data

**Figure 8:**
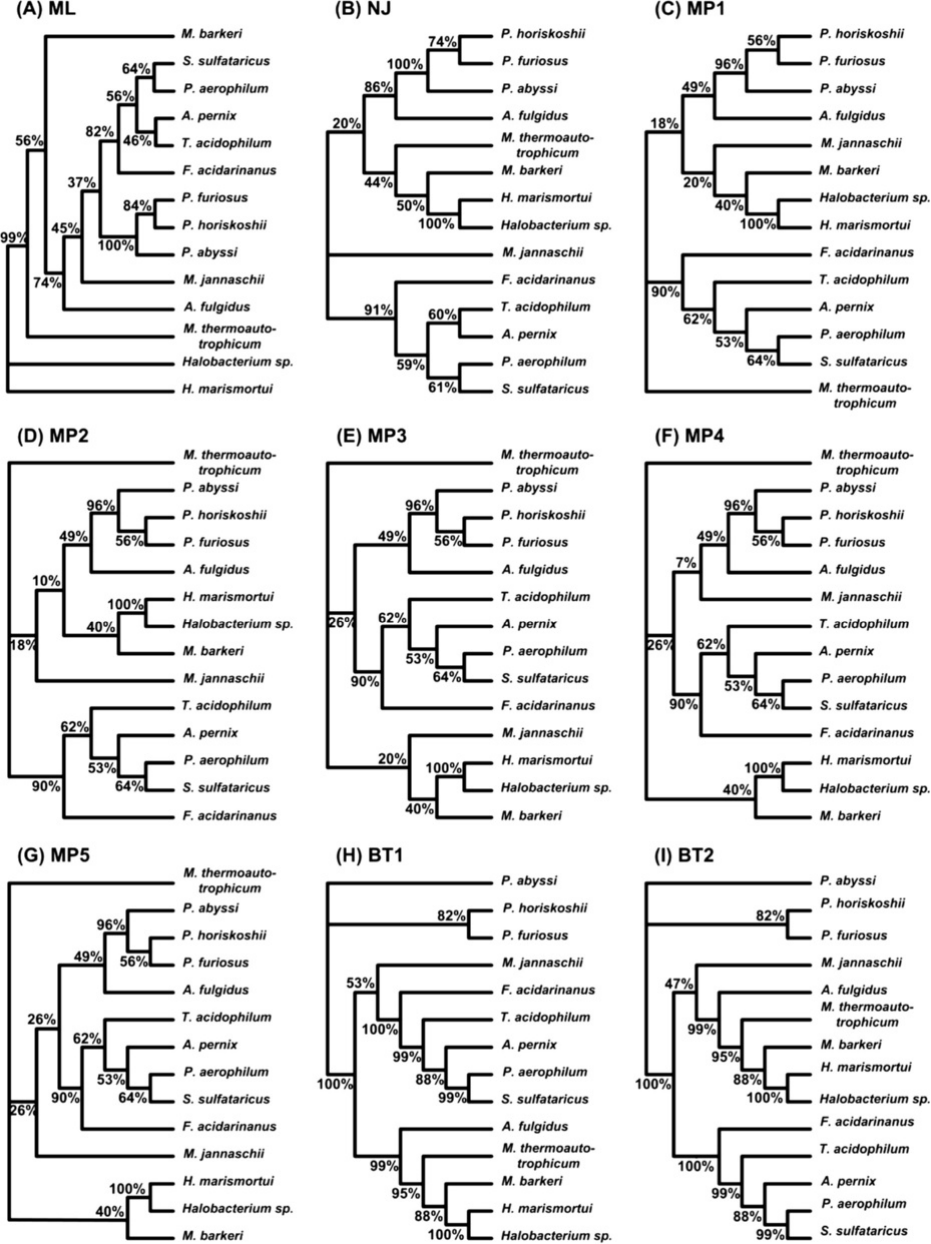
**The set of nine gene trees (A-I) inferred for the Archaebacteria dataset.** The abbreviations used in Figure 4 also apply here.

**Figure 9:**
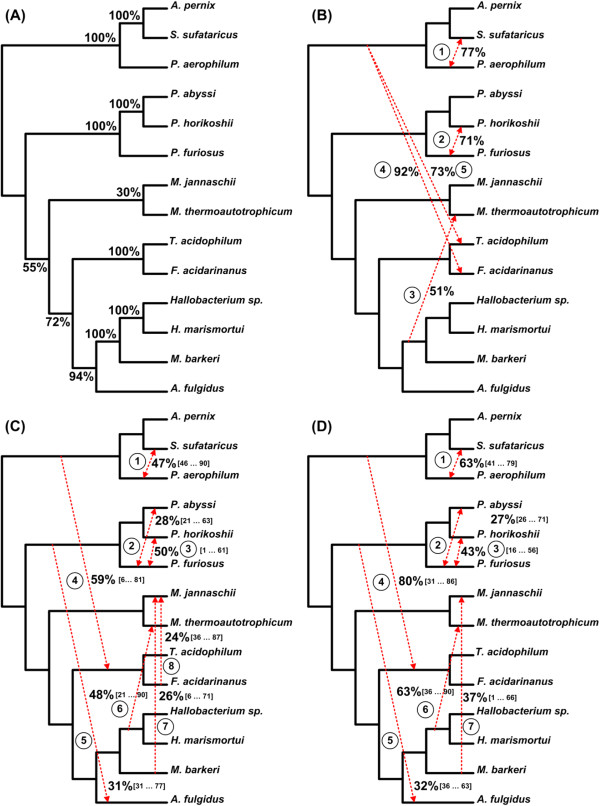
**Explicit weighted consensus horizontal gene transfer networks inferred for the Archaebacteria dataset. A)** species tree obtained by Matte-Tailliez et al. [49]; **B)** network obtained from full-length sequences using all the nine gene trees from Figure 8 (which were inferred using the ML, NJ, MP and BT approaches) and depicting complete horizontal gene transfer events; **C)** network obtained by the sliding window procedure with a ML method used for tree inference and depicting complete and partial horizontal gene transfers; **D)** network obtained by the sliding window procedure with a Bayesian method used for tree inference and depicting complete and partial horizontal gene transfers. The sequence interval corresponding to each partial horizontal gene transfer (see parts **C** and **D** of the figure) is given between brackets. The transfer number corresponds to its order of appearance in the gene transfer scenario found by our method. The bootstrap scores of internal branches of the species (backbone) tree and the weights of horizontal gene transfers are also indicated.

Similar to the two previous examples we used the four above-mentioned tree inference methods to build multiple phylogenies of the gene *rpl12e* for 14 Archaebacteria species originally analyzed by Matte-Tailliez et al. [49]. Thus, one maximum likelihood (Figure 8A), one distance-based (Figure 8B), five maximum parsimony (Figures 8C to 8G) and two Bayesian phylogenies (Figures 8H and 8I) were obtained. Considering the species tree (Figure 9A), which was reconstructed using the concatenation approach [49], we applied Algorithm 3 to the obtained phylogenies to infer a horizontal gene transfer network of the gene *rpl12e*. The species tree was used as the backbone topology to which we added the highly ranked incompatible clusters to build the weighted consensus evolutionary network encompassing a scenario of horizontal transfers of *rpl12e*. Using the cut-off level of 30%, we obtained five reticulation branches depicting alternative evolutionary histories (Figure 9B). Then, applying the above-discussed strategy for determining horizontal gene transfer direction (see function *find*_*direction*), we assigned directions to all obtained gene transfer branches. In the case of Transfers 1 and 2 (Figure 9B), the transfer direction cannot be retraced without discrepancy because both concurrent transfers are symmetric and lead to the same tree topology.

Note that in Figures 5A and 7A the supporting weights calculated by our method for the backbone and reticulation branches are given in percentages. For the network presented in Figure 9B our method was carried out to calculate the supporting weights of the reticulation branches only, whereas the weights of the internal branches of the backbone (species) phylogeny are the bootstrap scores provided by Matte-Tailliez and colleagues [49].

### Simulation results

**Figure 10:**
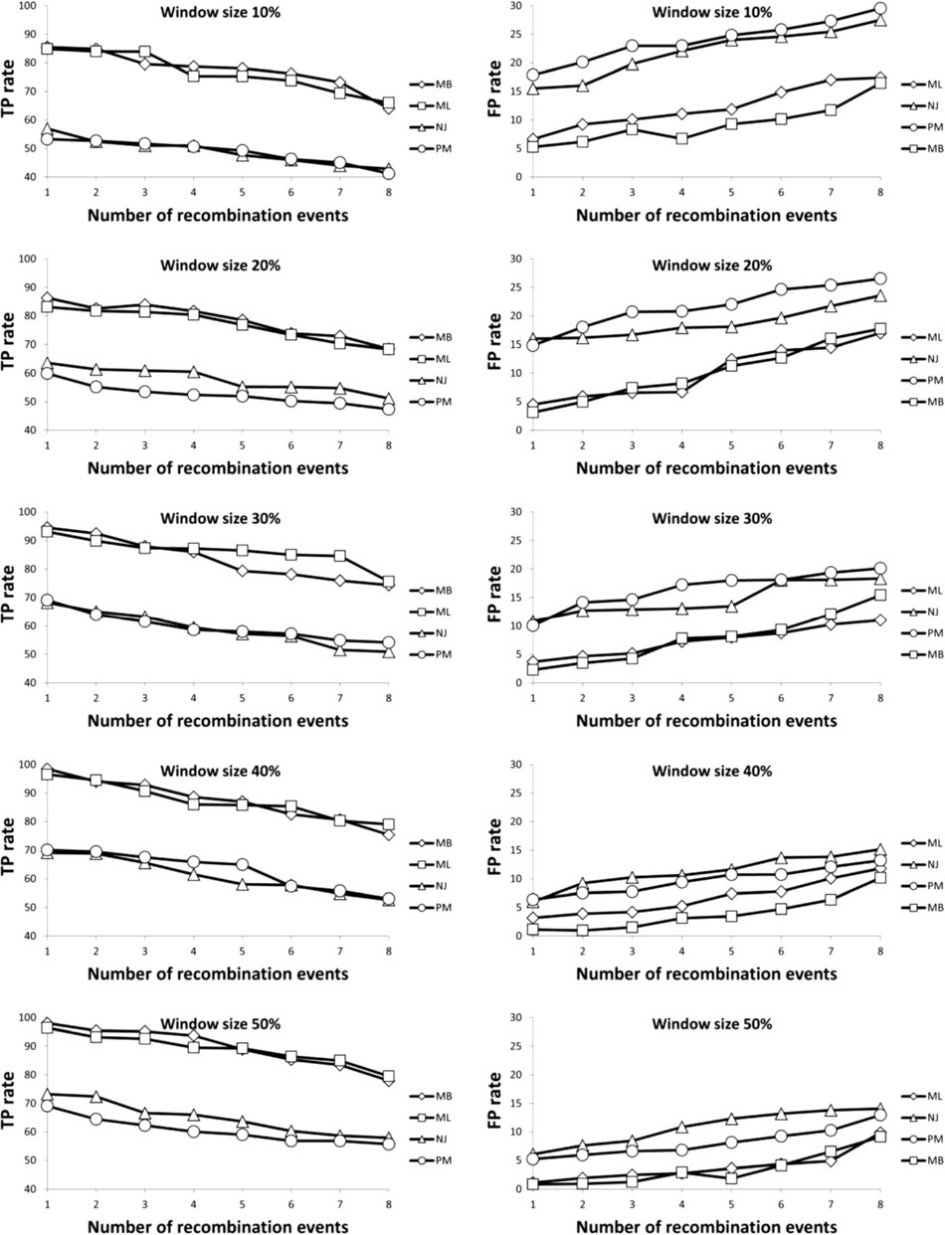
**Average true-positive (left-hand panel) and false-positive (right-hand panel) rates provided by the weighted consensus network reconstruction method depending on the number of recombination events in the simulated data and the tree inference method used.** The presented rates are the averages computed for different sliding window sizes (varying from 10 to 50% of the total MSA length) and different numbers of taxa (ranging from 16 to 64 with the step of 8); 100 datasets were tested for each parameter combination; ML, NJ, MP and BT abbreviations stand for the PhyML, BIOINJ, DNAPARS and MrBayes methods, respectively.

**Figure 11:**
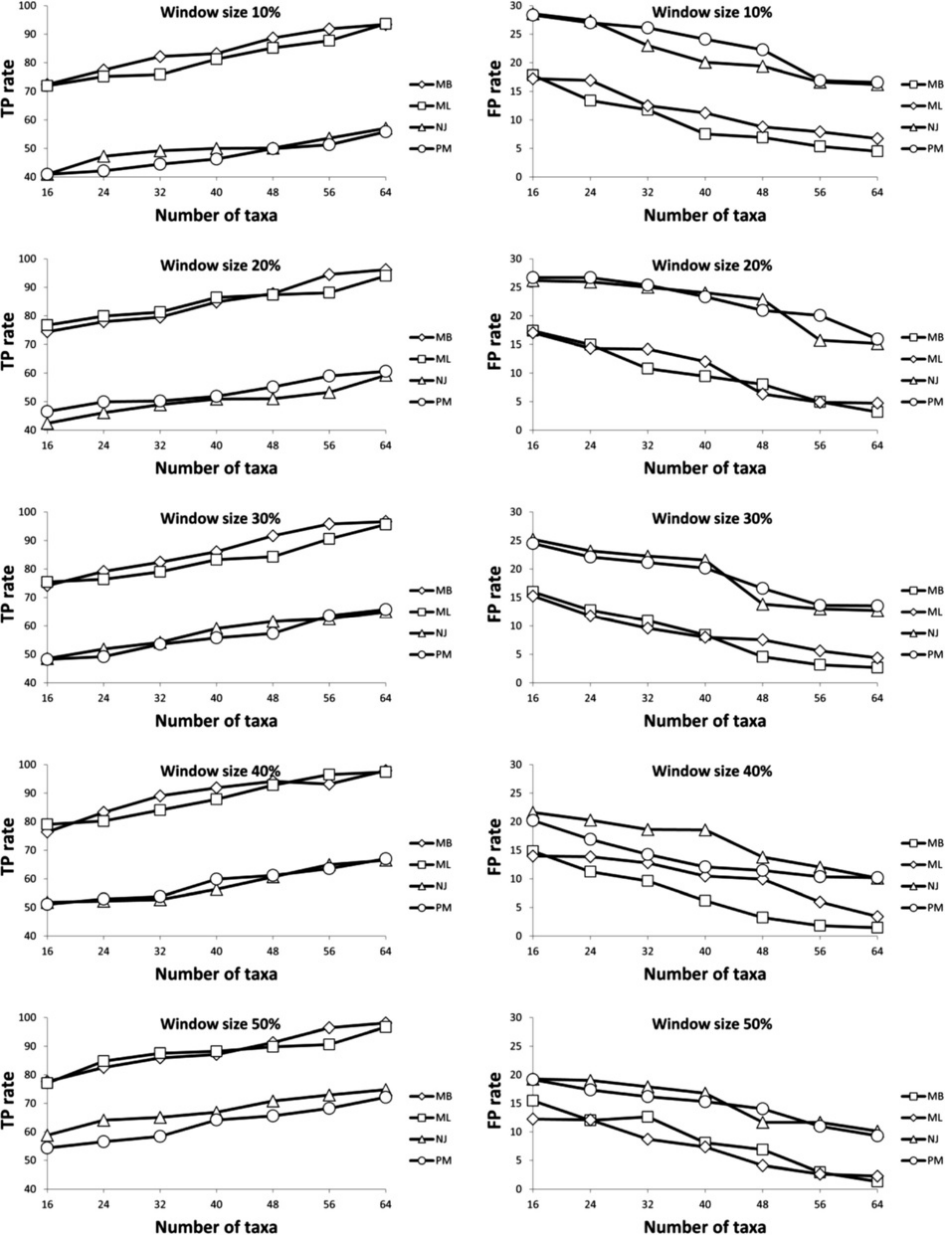
**Average true-positive (left-hand panel) and false-positive (right-hand panel) rates provided by the weighted consensus network reconstruction method depending on the number of taxa in the simulated data and the tree inference method used.** The presented rates are the averages computed for different sliding window sizes (varying from 10 to 50% of the total MSA length) and different numbers of recombination events (ranging from 1 to 8); 100 datasets were tested for each parameter combination; ML, NJ, MP and BT abbreviations stand for the PhyML, BIOINJ, DNAPARS and MrBayes methods, respectively.

The results provided by Algorithm 2 (inference of recombination events using a sliding window approach) on simulated data are shown in Figures 10 and 11. For each parameter combination, including the number of taxa, number of reticulation events and sliding window size, 100 datasets were generated and analyzed. The average rates of true and false positives characterizing our weighted consensus network building method are illustrated. Since in our simulations we knew the exact source and target of each reticulation event, we were able to estimate the success and failure rates of the consensus network method in terms of true positives and false positives by measuring the proportion of times when our method was able to identify both the exact source branch and destination branch of the event (i.e., true positive reticulation) and when either the source or destination branch of the detected event, or both of them, were different from the simulated ones (i.e., false positive reticulation). The *x*-axis depicts either the number of recombination events introduced in the data (Figure 10) or the number of taxa (i.e., number of species or tree leaves - Figure 11). The results obtained for the sliding windows whose width was equal to 10, 20, 30, 40 and 50% of the total length of the multiple sequence alignment are illustrated in different panels. The *y*-axis represents the average number of times when our weighted consensus network reconstruction method correctly (true positives - left-hand panels) or incorrectly (false positives - right-hand panels) identified intragenic recombination events.

The obtained results suggest that when the number of recombination events is small, they are more likely to be detected correctly. The best results in terms of both true and false positives were found for longer recombination fragments, i.e., 40 and 50% of the total length of the multiple sequence alignment. Another general trend is that the PhyML and MrBayes methods were much more effective in inferring the correct supporting tree and reticulation events than their BIONJ and DNAPARS counterparts. These results also suggest that it is much easier to detect recombination events in larger (i.e., 32 and 64-species) phylogenies. Furthermore, the probability of finding the correct reticulation events increases as the width of the sliding window becomes closer to the real length of the simulated recombination fragment.

### Searching for intragenic recombination and partial horizontal gene transfer events in real data

Considering the results obtained for simulated data, we applied Algorithm 2 based on the sliding window approach and the two best tree inference methods (PhyML and MrBayes) to reanalyze the honeybee, chloroplast and Archaebacteria data described above. The purpose of this new analysis was to discover alternative evolutionary events of smaller lengths (i.e., intragenic recombination and partial horizontal gene transfer events which trigger the formation of mosaic genes [62]). Those partial evolutionary events, in the sense that they concern only a part of the given gene might have gone unnoticed when analyzing the full-length gene sequences.

For the honeybee example, the PhyML and MrBayes methods allowed us to infer one and two possible recombination events (Figures 5B and 5C), respectively, in addition to a possible recombination event found in the analysis based on the full-length sequences (i.e., linking the species A. *mellifer* and A. *serana* in Figure 5A). For the chloroplast data, two additional reticulation events were detected using PhyML (Figure 7B) compared to the full-length sequence analysis (Figure 7A). Using MrBayes, we inferred four additional recombination events (Figure 7C) compared to the full-length sequence analysis three of which were concordant with the results obtained using PhyML.

For the smaller-scale recombination events found using Algorithm 2 for the honeybee and chloroplast data, the intervals where they were detected are indicated between brackets in addition to their supporting weights (see Figures 5B and 5C, Figures 7B and 7C). For the full-sequence analysis events found using Algorithm 1 (see Figures 5A and 7A), no intervals are given because the latter events apply to entire genes.

Finally, in the case of the Archaebacteria data, the PhyML and MrBayes methods allowed us to detect eight and seven partial horizontal gene transfers, respectively (Figures 9C and 9D). Three of the detected partial gene transfers (Transfers 1, 3 and 6 in Figures 9C and 9D), which were found by both methods, were also reported by Boc et al. [63] (a study dedicated to the detection of complete horizontal gene transfers) and Boc et al. [64] (a study dedicated to the detection of partial horizontal gene transfers). Two other partial gene transfers (Transfers 5 and 8 in Figure 9C) detected using PhyML (one of them was also detected using MrBayes; Transfer 5 in Figures 9C and 9D) were reported only in [64], while another gene transfer (Transfer 4 in Figures 9C and 9D) detected using both PhyML and MrBayes was a combination of two separate complete gene transfer events (Transfers 3 and 4 in Figure 9B) originally detected by Boc et al. [63]. Our method also identified two additional partial horizontal gene transfers (Transfers 2 and 7 in Figures 9C and 9D) that were not indicated in Boc et al. [64].

For comparison purposes, we also inferred splits graphs and cluster networks for the three above-mentioned real datasets using the SplitsTree [4,43] and Dendroscope [65] programs, respectively. Moreover, reticulograms were inferred for the honeybee and chloroplast datasets and a horizontal gene transfer network was constructed for the Archaebacteria dataset, both using the T-REX web server [63,66]. The NeighborNet algorithm [67] from the SplitsTree 4 software was used with the ordinary least-square optimization and convex hull algorithm options. The Dendroscope program [65] was carried out with the default parameters and the percent threshold equal to 20 to build cluster networks. The reticulogram inference algorithm was carried out using the weighted least-square method MW with global optimization [68] to infer the support tree and the stopping criterion *Q*_1_[3]. The HGT-Detection algorithm was performed with the HGT bootstrap option and the species and gene tree roots selected as described in [63].

**Figure 12:**
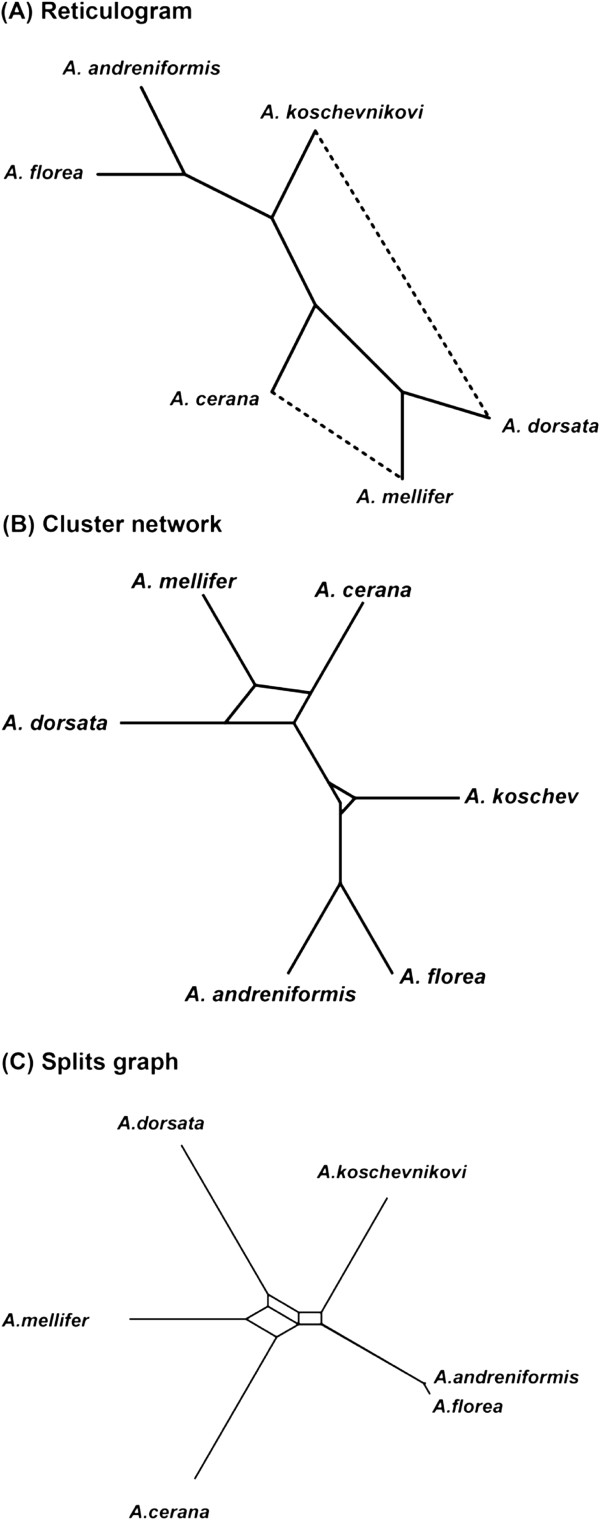
**Alternative network representations of the honeybee dataset.** They include: **A)** reticulogram obtained using the Reticulogram building algorithm from the T-REX web server; **B)** cluster network obtained by the Cluster network algorithm from the Dendroscope program; **C)** splits graph obtained by the NeighborNet algorithm from SplitsTree 4.

**Figure 13:**
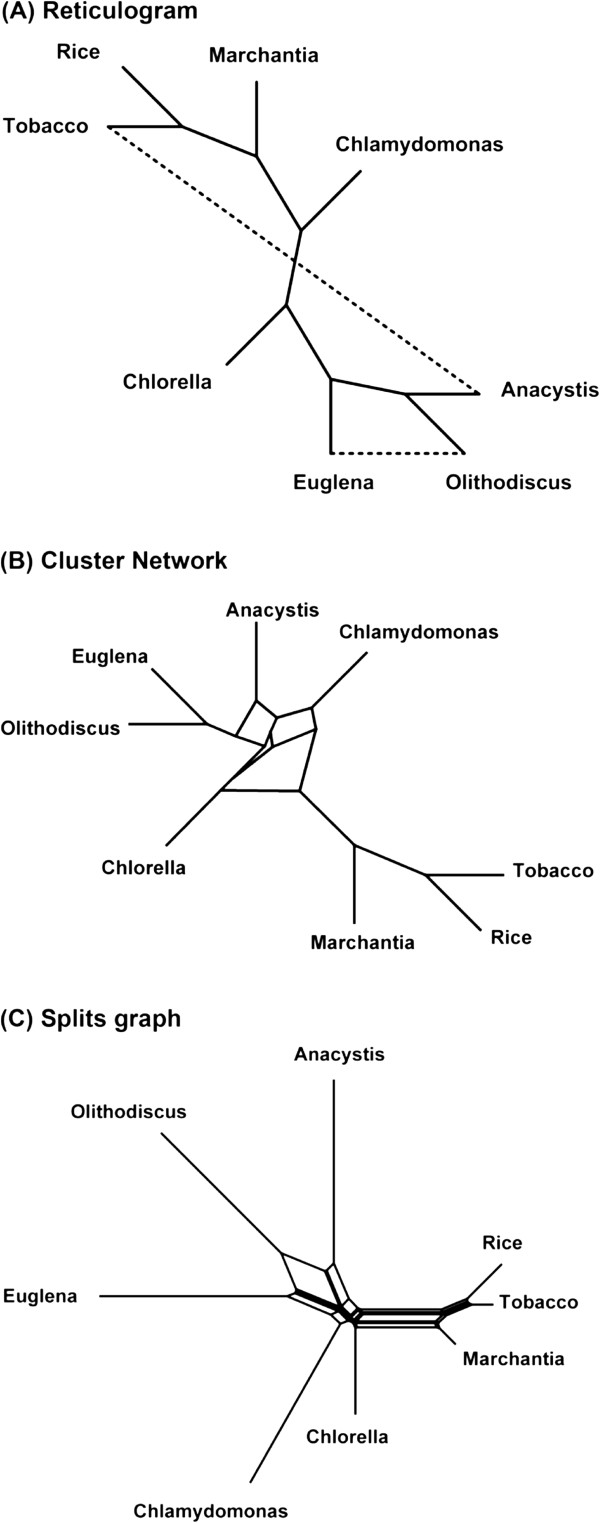
**Alternative network representations of the chloroplast dataset.** They include: **A)** reticulogram obtained by the Reticulogram building algorithm from the T-REX web server; **B)** cluster network obtained by the Cluster network algorithm from the Dendroscope program; **C)** splits graph obtained by the NeighborNet algorithm from SplitsTree 4.

**Figure 14:**
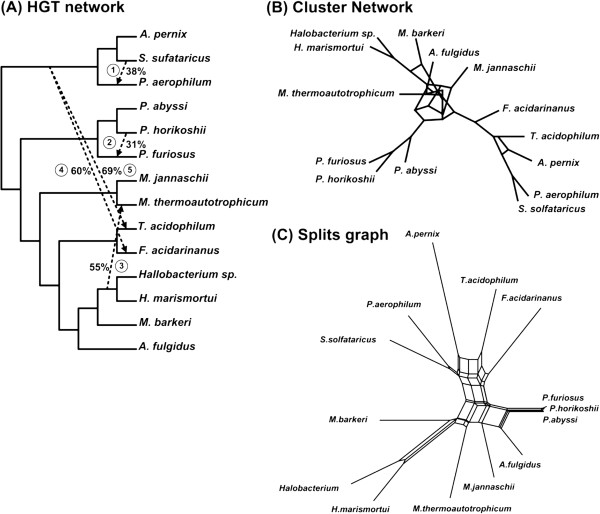
**Alternative network representations of the Archaebacteria dataset.** They include: **A)** horizontal gene transfer network obtained by the HGT-Detector algorithm from the T-REX web server; **B)** cluster network obtained by the Cluster network algorithm from the Dendroscope program; **C)** splits graph obtained by the NeighborNet algorithm from SplitsTree 4.

The obtained network representations are shown in Figures 12, 13 and 14 for the honeybee, chloroplast and Archaebacteria examples, respectively. In Figures 12A and 13A, one of the reticulation branches (represented by dashed lines) found by the reticulogram inference method was also identified by our weighted consensus network building method (i.e., the reticulation branches between (1) *A. mellifer* and *A. cerana* in Figure 12A and between (2) Euglena and Olithodiscus in Figure 13A). The similarities between horizontal gene transfer network found by us and by HGT-Detection [63] (Figure 14A) will be discussed in detail in the next section.

## Discussion and conclusion

Dealing with multiple incompatible phylogenies inferred either through the use of different reconstruction methods or by including multiple genes in the analysis has been always a major issue in phylogenetics. The degree of uncertainty increases in line with the number of various phylogenies inferred for the same set of species [38]. The concatenation approach, which has been widely used as a solution to the single-gene phylogenies discordance problem, has been proven to lead to biased and misleading phylogenies in many practical situations [30-32]. For instance, Kubatko and Degnan [34] showed that when the internal branches of a species phylogeny are short (due to adaptive radiation, increased number of taxa from the same group or recent divergences), the concatenation approach usually reduces the accuracy of standard phylogenetic methods. The latter authors also suggested that bootstrap scores obtained from concatenated datasets tend to show moderate to strong support for incorrect trees [34]. In general, the main drawback of the concatenation approach lies in its flawed assumption that all the genes (and in a similar way, the whole genomes) have been subject to the same evolutionary processes at the same evolutionary rate, and consequently, no heterogeneity exists among the genes. Given the broad occurrence of heterogeneity among genes and the high number of phylogenetic mechanisms influencing their evolution, one can argue that in a considerable number of cases the concatenation approach will fail to infer a reliable congruent phylogenetic tree or network. Since incongruence increases with the number of genes included in the analysis, proposing as a final cohesive solution a single phylogeny reconstructed using either the concatenation or the consensus tree approaches is only an indication of ignoring phylogenetic conflicts, and consequently, ignoring many widespread evolutionary processes such as horizontal gene transfer, recombination, hybridization and deep coalescence, which play major roles in the evolution of many species.

When the heterogeneity among genes is due to reticulate evolution, phylogenetic networks should be used in place of traditional or consensus phylogenetic trees [3-5]. Phylogenetic networks are generalizations of phylogenetic trees intended to represent both speciation and reticulate evolutionary events characterizing the given group of genes and species explicit networks) or to display conflicting evolutionary signals present in the data (implicit networks). To address the gene trees discordancy issue, we described here a new weighted consensus network reconstruction method which is able to infer and validate statistically the dominant evolutionary history of species (consensus tree) as well as the alternative evolutionary scenarios (consensus reticulation events).

Two practical situations are possible: we are either in possession of a reliable species phylogeny or not. In the case when we have a reliable species tree (e.g., when tree topology is confirmed via the Tree of Life project), we can directly define it as the network support structure. Otherwise, averaging the tree clusters present in the given gene trees and using the consensus tree approach as the starting point for building the consensus network is a natural way of computing the support species tree structure in the absence of reliable additional information. The weights are used to take into account the tree cluster support when building an explicit phylogenetic network. The more gene trees we have, even when some of them are affected by different reticulation events, the more reliable the consensus network is. The most difficult practical situation for our method is when we have only a few gene phylogenies, most of which are affected by the same reticulation event. But there is no any network building method that will infer a correct explicit phylogenetic network is such a situation.

We use both the discrepancy between the gene tree topologies (i.e. between the gene tree clusters) and statistical support of the gene tree branches in order to indentify the consensus network branches and reticulation events. Bootstrap scores or posterior probabilities of the gene tree clusters are constantly used to compute weights and thus to validate the selected network braches. The acceptance of some of the clusters and rejection of the other is determined by comparing the cluster weights to a pre-defined threshold. Indeed, like any other phylogenetic method, bootstrapping has its own pitfalls [69]. However, in general, bootstrap scores and posterior probabilities are widely-accepted statistical estimates which have been proven very useful for assessing statistical robustness of phylogenetic trees.

Many studies supported by simulations advocate the use of probabilistic methods over distance- and parsimony-based approaches for inferring phylogenetic trees [12,60,70]. Our general conclusion supported by the simulation results is that phylogenetic networks should be preferably reconstructed using maximum likelihood or Bayesian approaches as well. However, in some cases in this study, we used all the four main tree reconstruction approaches since different phylogenetic assumptions, optimality criterions and nucleotide or amino acid substitution models augment the collective probability of finding potential evolutionary conflicts.

In our first example examining the evolution of six honeybee species, we discovered a possible reticulate evolutionary history, suggesting that *A. cerana* could be a closer relative of *A. mellifer*, compared to the backbone species phylogeny in which the closest relative of *A. mellifer* is *A. dorsata* (Figure 5A - network obtained from the full-length sequences). This finding was consistent with a possible hybridization/recombination hypothesis involving the ancestors of *A. cerana* and *A. mellifer*, which was first formulated by Makarenkov and colleagues [71]. Our weighted hybridization networks constructed using the sliding window procedure (Figure 5B-C) suggest explicitly that *A. cerana* is a possible hybrid of *A. mellifer* and *A. koschevnikovi* (see the arrows stemming from the *A. mellifer* and *A. koschevnikovi* branches and entering into the *A. cerana* branch). The opposite arrows entering into the *A. cerana* branch concern the intervals that have a very short overlap in both cases (Figures 5B and 5C) what suggests a possible recombination event. We cannot provide such an easy interpretation for the corresponding reticulogram, cluster network or splits graph (Figures 12A to 12C, respectively). Note that the backbone phylogeny we built using the bootstrap-based extended majority rule was consistent with the species phylogeny inferred in [68].

Similarly, the dominant evolutionary history (i.e., the backbone phylogeny) we inferred when analyzing the chloroplast dataset was consistent with the findings of Makarenkov and Legendre [11]. The most significant reticulation event depicted in the network obtained from the full-length sequences (it is represented by a double-headed arrow in Figure 7A showing that each of the involved species might be a parent of the other) suggests a closer relationship between Euglena and Olithodiscus (i.e., stemming from a possible hybridization event involving the ancestors of these species) compared to the dominant scenario in which Olithodiscus is the closest neighbour of Anacystis. The networks inferred using the sliding window procedure (Figure 7B-C) suggest in addition that Chlamydomonas might be a hybrid species whose possible parents include the ancestors of Anacystis and Euglena, and the common ancestor of Tobacco, Rice and Marchantia, and that Euglena might be a parent of Chlorella.

In the horizontal gene transfer example, we considered the maximum likelihood phylogeny of 14 Archaean species inferred by Matte-Tailliez and colleagues [49] using the gene concatenation approach. This tree played the role of the species tree, representing the dominant evolutionary history, in our analysis (Figure 9A). First, using multiple phylogenies of the gene *rpl12e* inferred using the BIONJ, DNAPARS, PhyML and MrBayes methods (Figure 8) and Algorithm 3, we identified five potential horizontal gene transfer relationships not accounted for by the backbone tree topology (Figure 9B). Our findings were consistent with the horizontal gene transfer hypothesis formulated by Boc et al. [62]. Four transfer branches we inferred (see Transfers 1, 2, 4 and 5 in Figure 9B) were equivalent to those obtained by Boc and colleagues (Figure 14A). Furthermore, the fifth horizontal gene transfer we found (Transfer 3 in Figure 9B) differs from Transfer 5 in Figure 14A only by the presence of *M. bakeri* in the cluster of the donor organisms.

While full-length multiple sequence alignments can be directly used for finding diploid hybridization and complete horizontal gene transfer events, we need to consider the alignment fragments in order to detect smaller-scale evolutionary events, such as intragenic recombination and partial horizontal gene transfer (i.e., in the latter case a horizontal gene transfer is followed by an intragenic recombination leading to the formation of a mosaic gene [62]). The sliding window approach described above was applied here to search for partial gene transfers. The weighted consensus network of partial horizontal gene transfers built using Algorithm 2 (Figure 9C and D) allowed us to detect successfully five of the seven partial transfers originally predicted by Boc et al. [64].

In terms of visualization and results interpretation, our explicit network model is easily explicable, while the interpretation of implicit network models (e.g., splits graphs, cluster networks and reticulograms) becomes extremely difficult when dealing with a high number of species or conflicting events (see Figures 10B-C, 11B-C and 12B-C). Methods and software developed by Huson [43], Legendre and Makarenkov [3], Holland et al. [42,72] and Huson and Rupp [44] are rather devised to infer and visualize incompatibilities among gene trees without precisely describing the underlying evolutionary events. In contrast, our explicit weighted consensus network inference method is capable of detecting and validating, through the use of the weight function, the following reticulate evolutionary events: diploid or polyploid hybridization (recombination at the chromosome level), intragenic recombination, complete horizontal gene transfer and partial horizontal gene transfer followed by intragenic recombination. In a recent attempt, Guénoche [73] developed a method to tackle the problem of conflicting evolutionary signals by finding multiple consensus trees instead of a network as a method for separating and representing the evolution of diverging genes. In the future, it would be interesting to verify whether this method could be extended to the inference of multiple consensus phylogenetic networks representing alternative evolutionary hypotheses. The computer program implementing our method is available for download at the following URL: http://www.info2.uqam.ca/~makarenkov_v/ConsensusNetwork.rar.

## Competing interests

The authors declare that they have no competing interests.

## Authors’ contributions

ML and VM designed the methods. ML implemented them and carried out the simulations. PP-N and VM supervised the project and coordinated the development of the methods. All authors read and approved the final manuscript.
